# Health and Exercise

**Published:** 1886-10

**Authors:** 


### *HEALTH AND EXERCISE.*

We extract the following from a late issue of the *Journal of Education:*

When we are well—when the blood circulates freely, and every organ in the body is in good working order—what a luxury it is to live! How replete with interest is our work! What fine results we secure, and how many lively encouragements come to us! 'Tis then that our belief in God and goodness is strong. The dark problems of evil do not menace us so hopelessly—there is an answer for them all. We have such a sympathy with nature, the very sky and earth seem friends. And in the joy of conscious usefulness and in kinship with the objects about us, how often in spirit, if not in fact, do we stretch out joyful hands, saying with Miss Jewett's "Country Doctor," "My God, I thank Thee for my future!"

A physician once attended a patient who was suffering from a severe fit of indigestion. He found the mind of the man in as great a chaos as his stomach. "Doctor," said the poor fellow, "I am wretched. The face of my Saviour is hidden from me. What shall I do?" "Oh, well," said the dear old doctor, easily, "don't worry; it's *there; it's there fast enough.* Got covered up, perhaps, as the sun does; but *it's there, all right.*" A few hours after, when the patient had been relieved, the doctor said, "Well, D——, how about the face of Christ now?" "Clear and shining as the day," was the enthusiastic answer; "you were right, doctor." "And the matter with you, sir, was your stomach," was the sly rejoinder.

If we carefully examine our mental states, we shall find that they rise or fall, become large and noble, or belting and cramped, as the body remains healthy and vigorous, or enfeebled and abnormal.

It is a comparatively easy thing to keep well; so easy, in fact, that hundreds will not see it. We have faith in the doctors, and expect that a drug will effect what we were too indolent to do by following the simplest laws of health. Like the faithless generation of which Christ speaks, we demand a sign, and will not follow the clear light which God gives to every man for his guidance. What are, then, some of the simplest rules, the following of which will secure the perfect action of mind and body we so desire?

1. THE MATTER OF EXERCISE.—There is nothing equal to a good, brisk walk of two or three miles—more even—in the open air. There are two times in the day when this is especially beneficial—in the morning, after a light breakfast, and before supper or dinner at night.

Indoor life makes us lazy; and when that feeling of weariness and languor steals over us we want to stay in the house, curled up in some easy chair. But this is a dangerous thing to do; this is the time of all times when we ought to be out of doors exercising vigorously. What if it does tire us? It will be a healthy weariness from which we may soon recover, and which is far preferable to that nerve weariness which drives all hope of rest far away.

Riding and driving are good exercise, but hardly to be compared with walking. Practice walking suitably dressed, and with thick-soled boots, beginning with a short distance and gradually increasing it. Even those delicate in body may finally be able to accomplish, very readily, half-a-dozen miles on a stretch, with short and frequent rests.

The writer was one of thirteen persons, during the last summer, who enjoyed a 120-mile tramp through the White Mountain region. Of course there were tired and sore feet, sometimes, as well as other minor discomforts, but every member of the party gained through that experience so much health and enjoyment that walking in all weathers has ceased to be a bugbear.

Our English friends are far ahead of us in this respect. Would it not be well to follow suit? We may not be able to join the Appalachian Club, but we can form clubs of our own—clubs of one, if need be.

Along with the matter of walking comes that of other forms of physical exercise. Walking developes the lower part of the body much more than the upper. Some form of light gymnastics is necessary to strengthen the chest and arms. Dumb-bells or clubs will accomplish this. Better still, Dr. Forrest's "Home Exercises." This is simply a number of weights fastened to a rope running over a pulley, with handles attached. With this arrangement all the movements of lifting, pulling in and out, the "swimming stroke," etc., can be easily practised. Twenty minutes with this, twice a day, will produce a wonderful effect for good. This exercise is especially good on stormy days when walking must be omitted.

Much more might be said on the question of exercise. It is not only a good thing, but a vital necessity, if the individual would be well.

